# A Genome-Scale Metabolic Model for the Human Pathogen *Candida Parapsilosis* and Early Identification of Putative Novel Antifungal Drug Targets

**DOI:** 10.3390/genes13020303

**Published:** 2022-02-05

**Authors:** Romeu Viana, Diogo Couceiro, Tiago Carreiro, Oscar Dias, Isabel Rocha, Miguel Cacho Teixeira

**Affiliations:** 1 Department of Bioengineering, Instituto Superior Técnico, University of Lisbon, 1049-001 Lisboa, Portugal; romeuviana@tecnico.ulisboa.pt (R.V.); d.alexandre963@gmail.com (D.C.); tiagomslc@gmail.com (T.C.); 2 iBB—Institute for Bioengineering and Biosciences, 1049-001 Lisboa, Portugal; 3 Associate Laboratory Institute for Health and Bioeconomy—i4HB, 1049-001 Lisboa, Portugal; 4 CEB—Centre of Biological Engineering, Universidade do Minho, 4710-057 Braga, Portugal; odias@deb.uminho.pt; 5 ITQB Nova—Instituto de Tecnologia Química e Biológica António Xavier, 1049-001 Lisboa, Portugal; irocha@unl.pt

**Keywords:** *C. parapsilosis*, genome-scale metabolic model, drug target, drug discovery

## Abstract

*Candida parapsilosis* is an emerging human pathogen whose incidence is rising worldwide, while an increasing number of clinical isolates display resistance to first-line antifungals, demanding alternative therapeutics. Genome-Scale Metabolic Models (GSMMs) have emerged as a powerful in silico tool for understanding pathogenesis due to their systems view of metabolism, but also to their drug target predictive capacity. This study presents the construction of the first validated GSMM for *C. parapsilosis*—iDC1003—comprising 1003 genes, 1804 reactions, and 1278 metabolites across four compartments and an intercompartment. In silico growth parameters, as well as predicted utilisation of several metabolites as sole carbon or nitrogen sources, were experimentally validated. Finally, iDC1003 was exploited as a platform for predicting 147 essential enzymes in mimicked host conditions, in which 56 are also predicted to be essential in *C. albicans* and *C. glabrata*. These promising drug targets include, besides those already used as targets for clinical antifungals, several others that seem to be entirely new and worthy of further scrutiny. The obtained results strengthen the notion that GSMMs are promising platforms for drug target discovery and guide the design of novel antifungal therapies.

## 1. Introduction

In a world of climate and social change, human susceptibility to microbial disease is increased. In particular, fungal infections have seen a significant rise in incidence worldwide since the 1980s, with *Candida* spp. accounting for the majority of cases [1]. Although *Candida albicans* is the most commonly isolated species from candidiasis patients, the 1990s saw a shift in incidence within the genus towards non-*Candida albicans Candida* species (NCAC) [2]. From these, *Candida parapsilosis* has seen one of the most significant increases, often surging as the second most common etiological agent of *Candida* spp. infections, subverting historical trends in species incidence and even outranking *C. albicans* in some European countries [3]. Non-geographically restricted and with a broad range of virulence factors, adding to *C. parapsilosis’* already complex pathogenicity, are both the rise in resistance to first-line antifungals and the intrinsically lower susceptibility to alternative therapies, such as azoles [4] and echinocandins [1], respectively. Thus, there is a strong need to develop new antifungal therapies and develop new research tools to understand the metabolism of pathogens and, if possible, to use metabolic impairment as an antifungal strategy.

Genome-Scale Metabolic Models (GSMMs) have emerged as a systems biology approach to tackle this issue [5]. GSMMs correspond to the in silico reconstructed metabolic network of a given organism [6] and, thus, enable a systems perspective of metabolism. In the little more than 20 years since the publication of the first model [7], GSMMs have proven their applicability and versatility, from guiding strain design in metabolic engineering to elucidating novel drug target discovery in molecular medicine [8]. In the past, GSMMs have been mostly associated with the metabolic engineering of microbial cell factories due to their potential to simulate global metabolic behaviour and provide clues for the optimisation of added-value compound production [9]. However, recent examples have demonstrated the potential of these models in the search for new drug targets in pathogenic organisms [8,10,11,12,13,14].

This work presents the first validated in silico genome-scale metabolic reconstruction of the human pathogen *C. parapsilosis*, iDC1003. This model is provided in the well-established SBML format and can easily be read in most metabolic engineering platforms, such as OptFlux [15] and COBRA [16]. A set of predicted essential genes and reactions common to other pathogenic *Candida* spp. was obtained from the validated model, and their targetability as putative novel antifungal drug targets is discussed.

## 2. Materials and Methods

### 2.1. Model Construction

The herein described metabolic model reports on the yeast *Candida parapsilosis* with the taxonomic ID 5480. Model reconstruction was performed using *merlin* 4.0.5 [17], and further curation and validation were performed on OptFlux 3.0 [15] using the IBM CPLEX solver. Throughout the curation process, reactions were edited, manually added to, or removed from the model to correct gaps in the network using KEGG pathways, MetaCyc Database, and literature data as standards.

#### 2.1.1. Enzyme and Reaction Annotation

The initial draft model construction comprised enzyme and subsequent reaction annotation. The genome sequence of the reference strain *Candida parapsilosis CDC317* was obtained from NCBI’s Assembly database, accession number ASM18276v2 [18], and the Taxonomy ID from NCBI [19], which is required by merlin to identify the organism under study throughout the reconstruction process univocally. The genome-wide functional annotation was processed by *merlin* based on taxonomy and frequency of similar sequences through remote Basic Local Alignment Search Tool (BLAST) [20] similarity searches to the UniProtKB/Swiss-Prot database [21]. Hit selection was performed as described elsewhere [22], and phylogenetic proximity was implemented as described in Tsui et al. 2008 [23]. Protein–reaction associations available in the Kyoto Encyclopedia of Genes and Genomes (KEGG) [24] database were used to assemble the draft network.

#### 2.1.2. Correcting Reaction Reversibility, Directionality, and Balance

The initial reversibility curation was automatically performed by *merlin*, which implements information from remote tools such as eQuilibrator [25], as described by Dias et al. [22]. Further curation was entirely manual and justified, resorting to information from MetaCyc [26] and existing literature. Unbalanced reactions were identified automatically, and balancing was performed manually and based on MetaCyc [26], ChEBI [27], Brenda [28], and existing literature. All the reactions manually edited during the curation process can be found in Supplementary file S1. 

#### 2.1.3. Compartmentalisation

Compartmentalisation was implemented using WoLF PSORT [29], a protein localisation predictor, on the already connected non-compartmentalised model to simplify issue solving. The constructed model includes four compartments: extracellular, cytoplasm, mitochondrion, and peroxisome and one intercompartment: the cytoplasmic membrane.

A compartmentalised model calls for the implementation of transport reactions to connect intercompartment pathways. Transport reactions were generated using genomic information, together with the public database TCDB [30], using *merlin*’s integrated tool TranSyT [31]. Transport reactions across internal and external membranes for currency metabolites, such as H_2_O, CO_2_, and NH_3_, often carried by facilitated diffusion, were added to the model with no gene association.

#### 2.1.4. Defining the Biomass Equation

The biomass equation encompasses the cells’ major components and their relative numerical contributions—DNA, RNA, carbohydrates, lipids, and proteins—and acted as the objective function in the presented essentiality analysis. The content of each component was determined based on the literature. All the calculations were performed as described previously [32].

The reconstructed model also includes ATP requirements for both biomass production and cell maintenance—growth-associated maintenance (GAM) and non-growth-associated maintenance (NGAM), respectively. A GAM value of 25.65 mmol ATP/gDCW was considered for the biomass equation, calculated based on the ATP requirements for the biosynthesis of cell polymers, as shown in Mishra et al. [33], then adjusted for the considered biomass macromolecule composition.

Non-growth-associated ATP maintenance, the amount of ATP required by the cell repair and similar processes, was implemented in this model as an autonomous equation, thus forcing a basal ATP consumption—flux bounds inferred from *Candida tropicalis* [33]. The biomass equation’s components and their relative content are shown in Supplementary file S1.

The theoretical ratio used in the *S. cerevisiae* iMM904 metabolic model for the phosphorus-to-oxygen ratio was applied. Three generic reactions contributing to this ratio were automatically generated by *merlin* and were updated to replicate the same ratio as in the iMM904 model:

Reaction R00081:

1.0 Oxygen_mito_ + 4.0 Ferrocytochrome c_mito_ + 6.0 H^+^_mito_ ↔ 2.0 H_2_O_mito_ + 4.0 Ferricytochrome c_mito_ + 6.0 H^+^_cyto_, (1)

Reaction T02161:

1.0 Ubiquinol_mito_ + 2.0 Ferricytochrome c_mito_ + 1.5 H^+^ _mito_ ↔ 1.0 Ubiquinone_mito_ + 2.0 Ferrocytochrome c_mito_ + 1.5 H^+^_cyto_ (2)

Reaction T00485:

1.0 Orthophosphate_mito_ + 1.0 ADP_mito_ + 3.0 H^+^ _cyto_ ↔ 1.0 ATP_mito_ + 1.0 H_2_O_mito_ + 3.0 H^+^_mito_ (3)

The final balance reaction:

3.0 Orthophosphate_mito_ + 1.0 Oxygen_mito_ + 3.0 ADP_mito_ + 2.0 Ubiquinool_mito_ ↔ 3.0 ATP_mito_ + 5.0 H_2_O_mito_ + 2.0 Ubiquinone_mito_ (4)

### 2.2. Model Simulations and Enzyme Essentiality Prediction

The model simulations were performed using the flux balance analysis (FBA) [34] formulation on OptFlux 3.0 [15] using the IBM CPLEX solver. The determination of critical essential genes or reactions was performed with the following rationale: a gene/reaction is considered essential if, when removed from the model, this leads to a value of biomass flux of less than 5% of the reference value calculated for the wild-type strain. The essentiality of a gene/reaction was assessed by setting the flux of the reactions corresponding to a particular gene to zero and simulating the optimal growth rate with FBA. If deletion of one gene/reaction leads to non-growth, that gene/reaction is defined as essential. The simulations for gene/reaction essentiality were performed in environmental conditions simulating the RPMI medium, which mimics human serum.

### 2.3. Model Validation

#### 2.3.1. Strains and Growth Media

*C. parapsilosis* type strain *CDC317* was batch cultured at 30 ºC in orbital agitation (250 rpm) in yeast extract–peptone–dextrose (YPD) for inoculating cultivation and in synthetic minimal media (SMM) for growth parameter determination. Media compositions were as follows: YPD: 20 g/L glucose (Merck, Darmstadt, Germany), 20 g/L peptone (Merck, Darmstadt, Germany), and 10 g/L yeast extract (Merck, Darmstadt, Germany); SMM: 20g/L glucose (Merck, Darmstadt, Germany), 2.7 g/L ammonium sulphate (Merck, Darmstadt, Germany), 0.05 g/L magnesium sulphate (Riddle-de-Haen), 2 g/L potassium dihydrogen phosphate (Panreac, Barcelona, Spain), 0.5 g/L calcium chloride (Merck, Darmstadt, Germany), and 100 µg/L biotin (Sigma). 

#### 2.3.2. Aerobic Batch Cultivation

Precultures (100 mL) for aerobic batch experiments were grown in SMM in 500mL flasks at 30 ºC in orbital agitation (250 rpm). Cells were grown until the exponential phase and used to inoculate fresh media at an initial optical density at 600nm (OD_600nm_) of 0.3. Aerobic batch cultivations were incubated in SMM at 30 ºC with orbital agitation (250 rpm) for 10 h.

#### 2.3.3. Cell Density, Dry Weight, and Metabolite Concentration Assessment

During *C. parapsilosis* cell cultivation in SMM medium, 4 mL samples were harvested from the cell culture every two h, aiming for the quantification of biomass and extracellular metabolites. Cell density was monitored by measuring the OD_600nm_. For dry weight determination, culture samples were centrifuged at 13,000 rpm for 3 min and the pellets were lyophilised for 72 h at −80 °C and weighted. The supernatants were centrifuged once more for clarification and the concentrations of glucose, ethanol, glycerol, and acetic acid in the supernatants were determined by HPLC on an Aminex HPX-87 H Ion Exchange chromatography column, eluted with 0.0005 M H_2_SO_4_ at a flow rate of 0.6 mL/min at room temperature. Concentrations were determined through the corresponding calibration curves. Samples from any batch cultivation were analysed in triplicate. The specific growth rate, specific glucose consumption rate, and the specific production rates of ethanol, glycerol and acetic acid were calculated during the exponential growth phase as indicated elsewhere [35].

## 3. Results and Discussion

### 3.1. Model Characteristics, Highlighting C. Parapsilosis Unique Metabolic Features

The herein described *C. parapsilosis* metabolic model, iDC1003, comprises 1003 genes associated with 1804 reactions—of which 358 are drains (exchange constraints set to mimic the environmental conditions) and 536 are transport reactions—and 1278 metabolites across four compartments (extracellular, cytoplasm, mitochondria, and peroxisome) and within an intercompartment, the plasma membrane. The model can be found in an SBML format in Supplementary file S2.

Manual curation assessed a total of 847 reactions, from which 83 were mass balanced, 162 were corrected regarding reversibility or directionality, and 625 were added or removed from the model or had their annotation corrected or completed, as detailed in Supplementary file S1.

The biomass equation (Table 1) encompasses the cell’s major components, along with their respective and relative contributions—DNA, RNA, carbohydrate, protein, lipid, and cofactor content. The equation’s composition in carbohydrate [36], lipid [33,37]—sterol [33], phospholipid [33], and fatty acid [38]—and protein [33] was inferred from literature data. On the other hand, for the composition of DNA, the whole genome sequence was used to estimate the amount of each deoxyribonucleotide, as described in [39], while mRNA, rRNA, and tRNA were used to estimate the total RNA in the cell, as described in [9]. Essential metabolites were included in the biomass composition to account for the essentiality of their synthesis pathways 39 qualitatively. The growth and non-growth ATP requirements were inferred from Candida tropicalis [33].

**Figure 1:**
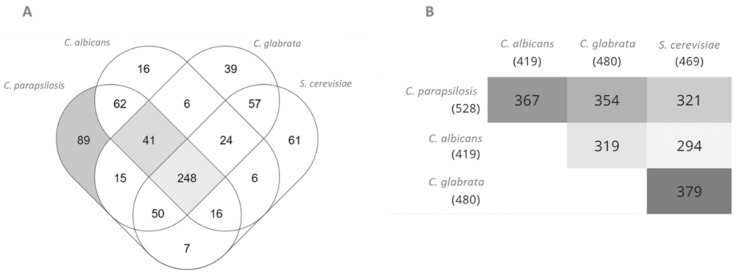
Comparison between *C. parapsilosis*, *C. albicans*, *S. cerevisiae*, and *C. glabrata* proteins with associated EC numbers present in the iDC1003, iRV781, iIN800, and iNX804 genome-scale metabolic models, respectively. (**A**): Venn diagram. (**B**): Pairwise intersections. Diagrams were obtained using Multiple List Comparator (www.molbiotools.com (accessed on 1 December 2021)).

The iDC1003 model was compared to the well-characterised genome-scale metabolic models of *C. glabrata* [40], *S. cerevisiae* [41], and *C. albicans* [14] to highlight unique metabolic features of *C. parapsilosis*. Although iDC1003 uses standard identifiers for reactions (KEGG ID), it is not possible to assess how the reactions differ among the four models, as the remaining models do not use the same identifiers (except for *C. albicans*). Thus, a comparison across the existing models was carried out based on the proteins associated with an EC number. After intersecting the EC numbers present in each of the three models, 85% of the proteins with an associated EC number in the *C. parapsilosis* model were found to also be present in at least one of the other three models (Figure 1). However, the remaining 15% (89/528) are exclusive to this model and may represent unique metabolic features of *C. parapsilosis*. It is also interesting to observe that 41 EC numbers are shared exclusively by Candida species and not by *S. cerevisiae*. These 41 enzymatic activities may be related to unique features of this genus, eventually linked to its pathogenicity. The complete list of unique EC numbers can be found in Supplementary file S1.

Occasionally, unique EC numbers might be related to outdated EC numbers or associated with other enzymes responsible for the same reactions in the different models. Nevertheless, specific cases stand out as potentially unique features of *C. parapsilosis*:

- 1.1.1.138: mannitol 2-dehydrogenase enables *C. parapsilosis* to use mannitol as a carbon source.

- 1.3.1.38: trans-2-enoyl-CoA reductase is involved in fatty acid elongation, likely affecting membrane properties.

- 3.1.4.12: sphingomyelin phosphodiesterase participates in sphingolipid metabolism, responsible for sphingomyelin hydrolysis.

- 3.5.1.75: urethane amidohydrolase enables *C. parapsilosis* to use urethane as a nitrogen source.

- 1.16.1.7: ferric-chelate reductase, which is involved in iron starvation, catalyses the reduction of bound ferric iron in a variety of iron chelators (siderophores), resulting in the release of ferrous iron.

- 1.16.3.1: ferroxidase, involved in iron homeostasis, oxidises Fe(II) to Fe(III), which allows the subsequent incorporation of the latter into proteins such as apotransferrin and lactoferrin.

### 3.2. Model Validation

#### 3.2.1. Assessing the Model’s Ability to Predict Carbon and Nitrogen Source Usage

Simulations were performed in SMM and compared to phenotypic growth data from existing literature to assess iDC1003’s reliability in predicting biomass production from different sole carbon or nitrogen sources. Data related to *C. parapsilosis* strains, other than the reference CDC317 strain, were also considered in the analysis to increase the number of carbon and nitrogen sources accounted for. A total of 47 sole carbon sources and 17 sole nitrogen sources were evaluated. The *C. parapsilosis* model correctly predicted growth in 94% of the carbon sources tested and in 100% of the nitrogen sources (Table 2). The model only failed for three carbon sources, 2-Keto-D-gluconic acid, L-arabinose, and ribitol, which the literature indicates that *C. parapsilosis* can use for growth. Interestingly, as far as it could be assessed, there is no experimental evidence of any enzymes behind these pathways existing in yeasts. It would be interesting to look deeper into these organisms’ eventually unique 2-Keto-D-gluconic acid, L-arabinose, and ribitol assimilation pathways.

#### 3.2.2. Assessing the Model’s Ability to Quantitatively Predict Growth Parameters

Specific growth rate, glucose consumption rate, and metabolite production rates were experimentally determined to validate the model quantitatively due to the lack of literature data for *C. parapsilosis*. *C. parapsilosis* CDC 317 cells were cultivated in SMM medium, and growth was followed by regular measurements of the OD_600nm_ and the cell dry weight. Samples were harvested to assess glucose concentration as a function of time during the exponential growth phase. In these conditions, a glucose consumption rate of 2.098 +/− 0.404 mmol·gDCW^−1^·h^−1^ was determined, while no ethanol, glycerol, or acetate production could be detected.

A simulation of the system’s behaviour in environmental conditions of SMM medium with a fixed glucose uptake flux of 2.098 mmol·g^−1^ dry weight·h^−1^ was performed to evaluate the model’s ability to predict the specific growth rate. The remaining nutrient fluxes were left unconstrained, as the system in these conditions is glucose-limited. Considering the experimentally determined glucose consumption rate of 2.098 mmol·gDCW^−1^·h^−1^, the model predicted a specific growth rate of 0.172 h^−1^. Compared to the experimentally determined rate of 0.159 +/− 0.027 h^−1^, the predicted growth rate is within the uncertainty interval of the experimentally determined parameter. Thus, there is no significant difference between both, which suggests iDC1003 can predict *C. parapsilosis* growth parameters (Table 3) quantitatively. Additionally, the formation of glycerol, acetic acid, and ethanol as byproducts was not predicted to occur, which agrees with the experimental data and the notion that *C. parapsilosis* is a Crabtree-negative yeast. Altogether, iDC1003 was proved to predict the main metabolic features of *C. parapsilosis* quantitatively.

### 3.3. Enzyme Essentiality Assessment: Looking for New Drug Targets 

Identification of essential enzymes of a given pathogen should, in principle, lead to the identification of good drug targets since, if one enzyme is essential for its growth or survival, a compound capable of inhibiting it could potentially be used as a drug with pharmacological activity against that pathogen. The drug target will be ideal if it is essential, or at least essential under conditions that mimic the human host environment, while having no human homolog. Based on these principles, iDC1003 was used to identify potential new drug targets by determining enzyme essentiality. For that, a list of essential reactions was obtained through simulation of the system’s behaviour in RPMI medium, which mimics the environmental conditions of human serum. A total of 147 enzymes were predicted as essential in iDC1003, excluding drains, transport reactions and those without an associated or incomplete EC number. The complete list of predicted essential enzymes can be found in Supplementary file S1.

**Figure 2:**
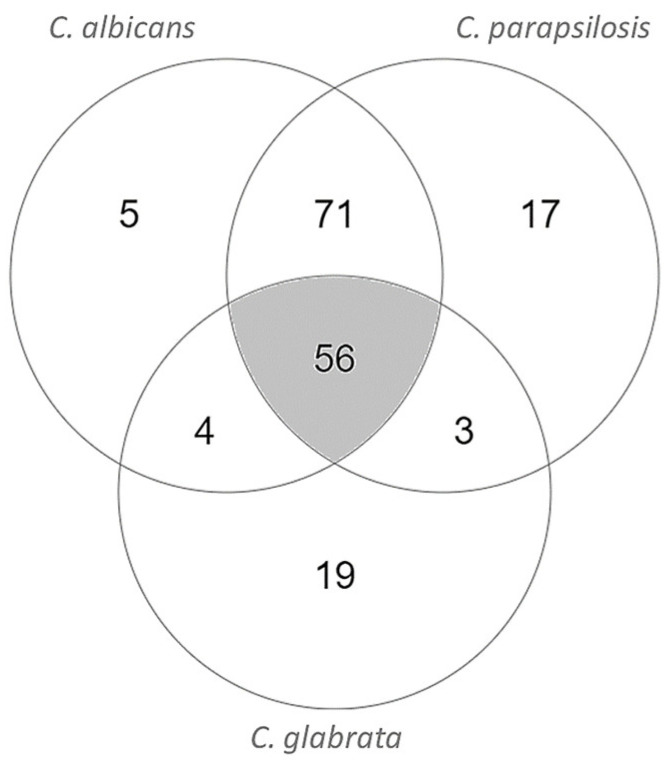
Intersection of *C. parapsilosis*, *C. albicans*, and *C. glabrata* essential EC numbers in RPMI medium environmental conditions in the genome-scale metabolic models iDC1003, iRV781, and iNX804, respectively. Diagrams were obtained using Multiple List Comparator (www.molbiotools.com (accessed on 1 December 2021)).

Finally, we decided to intersect the potential drug targets obtained through the *C. parapsilosis* model with those resulting from the two existing models for pathogenic *Candida* species, *C. albicans* [14] and *C. glabrata* [40], to focus on essential enzymes that can be used as targets in the treatment of infections caused by all *Candida* species. After the intersection, 56 essential enzymes common to the three models were found (Table 4, Figure 2).

Consistently, well-established antifungal drug targets were identified, including the well-known targets of azoles and echinocandins, Erg11 and Fks1, respectively. Additionally, some of the identified predicted drug targets are homologs of enzymes used as drug targets against other pathogenic organisms, including Imh3, which is targeted by inosinic acid in *Streptococcus pyogenes*, Fol1, a target of sulfonamides, and Fas1, a target of ethionamide, used in the treatment against *Mycobacterium tuberculosis*. These confirmatory results illustrate the potential of the used approach in the quest for new drug targets. More interesting, however, is the identification of numerous potential targets, as identified herein, that are not currently targeted by any drug used in clinical practice. There are some new targets with tremendous potential, as these do not have an orthologous enzyme in humans. Although not an excluding factor, the absence of a human ortholog is a preferable attribute, as this translates into lower chances of host drug toxicity and may allow for greater freedom of drug design.

Among the identified potential new drug targets, Abz1/2, Erg4, and Ura1 emerge as enzymes without any human homolog or drug association.

**Figure 3:**
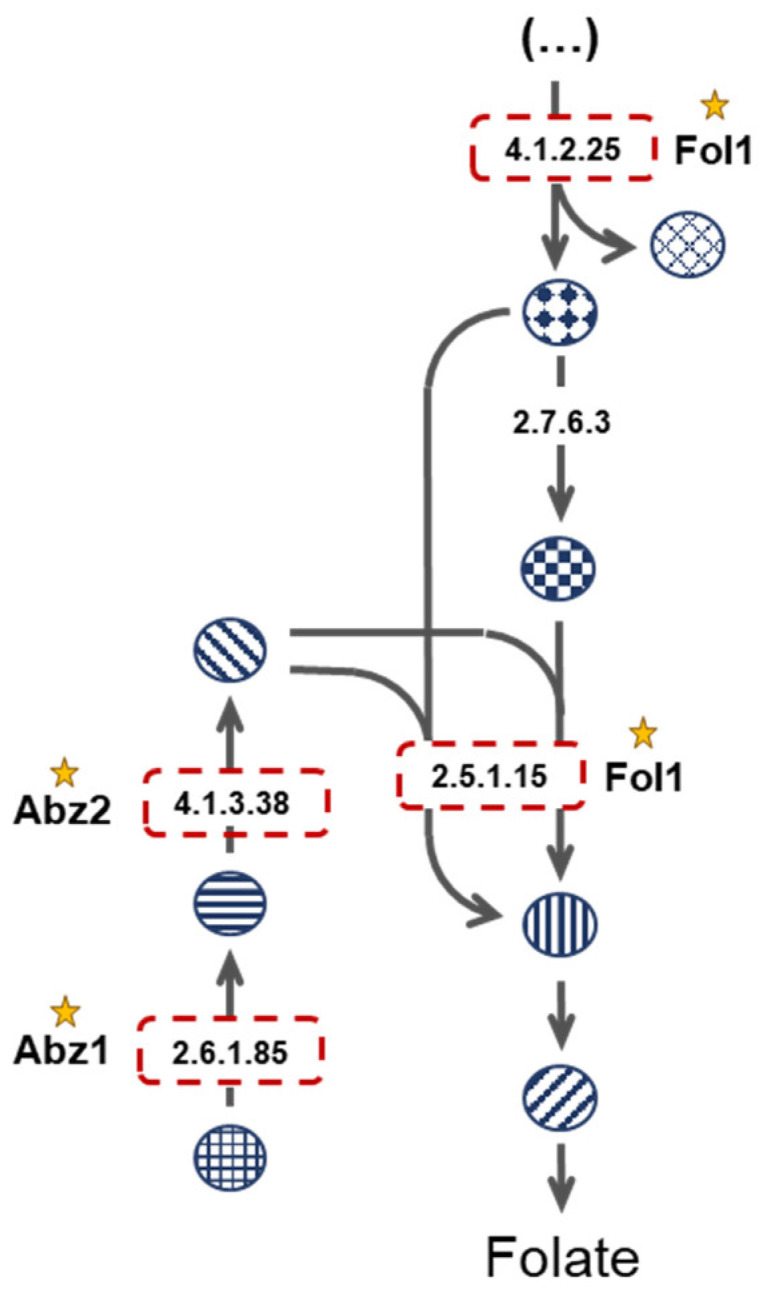
Folate biosynthetic pathway. Red boxes highlight the enzymes considered essential in the three analysed models.

Fungi rely on folate de novo biosynthesis given their inability to uptake folate from the environment [50]. *FOL1*, *ABZ1*, and *ABZ2* encode key enzymes in the folate biosynthesis pathway (Figure 3). Furthermore, these enzymes have no human orthologs, as humans rely on diet-derived folate [50]. The dihydropteroate synthase encoded by *FOL1* has been shown to be successfully inhibited by antifolates, such as sulfonamides, in a series of microorganisms [50,51]. However, antifolate therapy for *Candida* infections is not particularly effective considering current antifolate compounds [52]. In fact, for *C. albicans*, only sulfanilamide is used clinically, and it is restricted to topical use [53]. Given the efficacy of antifolates in treating infections by other etiologic agents, this might present the opportunity to design new effective antifungal compounds against *Candida* Fol1. On the other hand, no inhibitors of the para-aminobenzoate synthetase encoded by *ABZ1* or of the 4-amino-4-deoxychorismate lyase encoded by *ABZ2* are currently known, making these two enzymes fully novel putative drug targets worth further exploitation.

The Ura1 and Erg4 proteins may also be interesting drug targets. Ura1 has no human ortholog and, although this protein is not the target of any known drug, Aro9, from the same pathway, is a known target of atovaquone used to treat *Plasmodium falciparum* infections. In turn, Erg4, also with no human ortholog, is involved in ergosterol biosynthesis, a pathway currently targeted by azole drugs. 

## 4. Conclusions

The first validated global metabolic model for the human pathogen *C. parapsilosis* is presented in this study. The model was manually curated and validated experimentally and proved to be able to predict the main metabolic features of *C. parapsilosis* quantitatively. Furthermore, iDC1003 is robust in predicting the use of several carbon and nitrogen sources. The model shares 85% of the proteins with an associated EC number in other published yeast models. However, 15% of them are exclusive to this model and may represent some unique metabolic features of *C. parapsilosis*. Using iDC1003, several enzymes were predicted to be essential in RPMI medium, including some already known targets of antifungal agents and other antimicrobial agents used in clinical practice, illustrating the potential of the used approach in the quest for new drug targets. Several of the identified potential drug targets are not currently targeted by any drug used in clinical practice, and deserve further study. Among the identified potential new drug targets, Abz1/2, Erg4, and Ura1 stand out as enzymes without any human homolog or drug association. However, all other targets are worthy of scrutiny as, in fact, many of the drugs currently used in clinical practice have human orthologs. In these cases, the strategy may involve taking advantage of the structural differences between the proteins in the two organisms to design efficient compounds.

Despite the clear usefulness of these types of models, it is important to highlight that these reconstructions have limitations. Firstly, the basis of GSMMs is the genome’s functional annotation. Depending on the stringency of the criteria imposed on hit selection, such a procedure may lead either to compromising rates of false positive or negative annotations. Furthermore, such models do not encompass regulatory processes due to the high complexity of such an integration. Note how enzymatic activity can be regulated at different levels—from gene expression to post-translational modifications—and may result in given pathways being preferential in certain environmental conditions. The exclusion of such mechanisms, particularly regarding essentiality prediction, may result in predicted essential ECs that would otherwise not be metabolically relevant in the conditions of interest. Lastly, these simulations do not consider supra-metabolic factors, such as stress factors. Even so, and considering all these limitations, GSMMs allow for increasingly guided and reliable drug target discovery—as illustrated in this paper.

## Figures and Tables

**Table 1:** Biomass composition used in the model iDC1003. The full individual validated contributions of each of these metabolites are shown in Supplementary file S1.

Metabolite	g/gDCW	Metabolite	g/gDCW
Lipids		Proteins	
Lanosterol	0.00063	L-Valine	0.03536
Squalene	0.00017	L-Tyrosine	0.02771
Ergosterol	0.00455	L-Tryptophan	0.01356
Phosphatidylserine	0.00237	L-Threonine	0.02230
1-Phosphatidyl-D-myo-inositol	0.00173	L-Serine	0.02478
Phosphatidylcholine	0.00288	L-Proline	0.01902
Phosphatidylethanolamine	0.00194	L-Phenylalanine	0.02845
Phosphatidic acid	0.00052	L-Methionine	0.04275
Phosphatidylglycerol	0.00186	L-Lysine	0.06440
Tetradecanoic acid	0.00001	L-Leucine	0.03933
Hexadecanoic acid	0.00074	L-Isoleucine	0.02115
(9Z)-Hexadecenoic acid	0.00010	L-Histidine	0.01887
Octadecanoic acid	0.00032	L-Glutamate	0.03987
(9Z)-Octadecenoic acid	0.00278	L-Cysteine	0.00487
(9Z,12Z)-Octadecadienoic acid	0.00071	L-Aspartate	0.00346
(9Z,12Z,15Z)-Octadecatrienoic acid	0.00016	L-Asparagine	0.00362
Triacylglycerol	0.00467	L-Arginine	0.00008
Monoacylglycerol	0.00401	L-Alanine	0.03551
Diacylglycerol	0.00316	Glycine	0.02150
Sterol esters	0.00445	L-Glutamine	0.03987
**Soluble Pool**		**Carbohydrates**	
Thiamine	0.00096	Chitin	0.01170
Ubiquinone-6	0.00096	Mannan	0.23437
NADP+	0.00096	β (1,3)-Glucan	0.13621
NAD+	0.00096	**Deoxyribonucleotides**	
FMN	0.00096	UTP	0.01599
FAD	0.00096	GTP	0.01378
CoA	0.00096	CTP	0.01313
Biotin	0.00096	ATP	0.01730
Pyridoxal phosphate	0.00096	**Ribonucleotides**	
Tetrahydrofolate	0.00096	dTTP	0.00111
		dGTP	0.00074
		dCTP	0.00086
		dATP	0.00111

**Table 2:** In silico predictions versus in vivo described data regarding *C. parapsilosis*’ ability to grow in the presence of sole carbon and nitrogen sources. From the 62 different tested compounds, iDC1003 correctly predicted positive or null biomass production on 95%. A plus represents biomass production (+), a minus (−) no biomass production, and prediction disparities are highlighted in bold. Referenced data from Westerdijk fungal collection refer to strains CBS 1954 and CBS 604.

	In Vivo	In Silico	Reference		In Vivo	In Silico	Reference
Carbon Source							
Glucose	+	+	[42,43,44,45,46,47]	L-Sorbose	+	+	[47]
Maltose	+	+	[43,47]	D-arabinose	−	−	[43,47]
Sucrose	+	+	[42,44,45,46,47]	**L-arabinose**	**+**	**−**	[43,47]
Lactose	−	−	[43,45,46,47]	i-Erythritol	−	−	[43,47]
Galactose	+	+	[43,47]	Fucose	−	−	[43]
Melibiose	−	−	[43,44,46,47]	Salicin	−	−	[43,47]
Cellobiose	−	−	[43,47]	Arbutin	−	−	[43,47]
Inusitol	−	−	[43,46,47]	D-ribose	+	+	[43,47]
Xylose	+	+	[43,45,46,47]	D-Gluconate	+	+	[47]
Raffinose	−	−	[43,47]	**2-Keto-D-gluconic acid**	**+**	**−**	[47]
Trehalose	+	+	[43,47]	Inulin	−	−	[47]
Galactitol	−	−	[43,45,46,47]	D-Glucosamine	−	−	[47]
Rahmnnose	−	−	[43,47]	D-Galacturonate	−	−	[47]
Glycerol	+	+	[43,47]	Quinate	−	−	[47]
**Ribitol**	**+**	**−**	[43,47]	D-Glucono-1,5-lactone	+	+	[47]
Mannitol	+	+	[43,47]	Propane-1,2-diol	−	−	[47]
Sorbitol	+	+	[43,47]	D-Glucarate	−	−	[47]
Ethanol	+	+	[43,47]	L-Arabinitol	−	−	[47]
Methanol	−	−	[47]	D-Glucuronate	−	−	[47]
Succinate	+	+	[47]	Butane 2,3 diol	−	−	[47]
Lactate	−	−	[47]	D-Galactonate	−	−	[47]
Citrate	+	+	[47]	D-Tagaturonate	−	−	[47]
Starch	−	−	[47]	Fructose	+	+	[42]
Xylitol	+	+	[47]				
**Nitrogen Source**							
Ammonium	+	+	[47,48]	Urethane	+	+	[49]
Citrate	−	−	[47]	Creatine	−	−	[47]
L-Lysine	+	+	[47]	Imidazole	−	−	[47]
Creatinine	−	−	[47]	L-Glutamate	+	+	[48]
D-Tryptophan	−	−	[47]	L-Proline	+	+	[48]
Nitrite	−	−	[47]	L-Isoleucine	+	+	[48]
Cadaverine	+	+	[47]	Allantoin	+	+	[48]
Glucosamine	−	−	[47]	4-Aminobutanoate	+	+	[48]
Ethylamine	+	+	[47]				

**Table 3:** Growth parameters of iDC1003 and comparison with experimentally determined values.

	Specific Growth Rate (h^−1^)	q (mmol g^−1^ dry weight h^−1^)			
		Glucose	Ethanol	Glycerol	Acetic acid
In silico	0.172	2.098	0	0	0
In vivo	0.159 ± 0.027	2.098 ± 0.404	0	0	0

**Table 4:** Enzymes predicted to be essential in RPMI medium based on the screening of the genome-scale metabolic models of *C. parapsilosis*, iDC1003, *C. albicans*, iRV781, and *C. glabrata*, iNX804.

Gene Name			EC Number	Gene Name			EC Number
*C. Parapsilosis*	S. Cerevisiae	Human		*C. Parapsilosis*	S. Cerevisiae	Human	
	Homolog	Homolog			Homolog	Homolog	
*CPAR2_302110*	*ERG26*	*NSDHL*	1.1.1.170	*CPAR2_805350*	*PEL1*	*PGS1*	2.7.8.5
*CPAR2_104580*	*IMD4*	*IMPDH*	1.1.1.205	*CPAR2_804250*	*PHO8*	*ALPL*	3.1.3.1
*CPAR2_801560*	*ERG27*	*DHRS11*	1.1.1.270	*CPAR2_602700*	*GEP4*	*PTPMT1*	3.1.3.27
*CPAR2_110330*	*HMG1*	*HMGCR*	1.1.1.34	*CPAR2_100500*	*URA4*	*CAD*	3.5.2.3
*CPAR2_405900*	*ERG24*	*TM7SF2*	1.3.1.70	*CPAR2_806200*	*IPP1*	*PPA2*	3.6.1.1
*ERG4*	*ERG4*	*-*	1.3.1.71	*CPAR2_805940*	*ADE2*	*PAICS*	4.1.1.21
*CPAR2_206550*	*TMP1*	*TYMS*	2.1.1.45	*URA3*	*URA3*	*UMPS*	4.1.1.23
*CPAR2_202250*	*ADE17*	*ATIC*	2.1.2.3	*CPAR2_109530*	*MVD1*	*MVD*	4.1.1.33
*CPAR2_203160*	*URA2*	*CAD*	2.1.3.2	*CPAR2_800750*	*CAB3*	*PPCDC*	4.1.1.36
*CPAR2_203160*	*URA2*	*CAD*	6.3.5.5	*CPAR2_303390*	*FOL1*	*-*	4.1.2.25
*CPAR2_106400*	*FKS1*	*-*	2.4.1.34	*CPAR2_303390*	*FOL1*	*-*	2.5.1.15
*CPAR2_802790*	*URA5*	*UMPS*	2.4.2.10	*CPAR2_212310*	*ABZ2*	*-*	4.1.3.38
*CPAR2_208260*	*ADE4*	*PPAT*	2.4.2.14	*CPAR2_204960*	*ADE13*	*ADSL*	4.3.2.2
*CPAR2_302840*	*BTS1*	*GGPS1*	2.5.1.1	*CPAR2_401630*	*IDI1*	*IDI1*	5.3.3.2
*CPAR2_103950*	*ERG20*	*FDPS*	2.5.1.10	*CPAR2_301800*	*ERG7*	*LSS*	5.4.99.7
*CPAR2_403110*	*ABZ1*	*-*	2.6.1.85	*CPAR2_212740*	*MET7*	*FPGS*	6.3.2.17
*CPAR2_502760*	*CAB5*	*COASY*	2.7.1.24	*CPAR2_500190*	*ADE1*	*PAICS*	6.3.2.6
*CPAR2_202590*	*FMN1*	*RFK*	2.7.1.26	*CPAR2_208400*	*ADE5,7*	*GART*	6.3.3.1
*CPAR2_602050*	*CAB1*	*PANK*	2.7.1.33	*CPAR2_208400*	*ADE5,7*	*GART*	6.3.4.13
*CPAR2_105320*	*URA6*	*CMPK2*	2.7.4.14	*CPAR2_803640*	*ADE12*	*ADSS*	6.3.4.4
*CPAR2_400710*	*ERG8*	*PMVK*	2.7.4.2	*CPAR2_204070*	*ADE6*	*PFAS*	6.3.5.3
*CPAR2_304260*	*PRS1*	*PRPS1*	2.7.6.1	*CPAR2_804060*	*ACC1*	*ACACA*	6.4.1.2
*CPAR2_500260*	*PIS1*	*CDIPT*	2.7.8.11	*CPAR2_803530*	*ERG12*	*MVK*	2.7.1.36
*CPAR2_211620*	*ADE8*	*GART*	2.1.2.2	*CPAR2_701400*	*ERG13*	*HMGCS*	2.3.3.10
*CPAR2_602300*	*COQ3*	*COQ3*	2.1.1.114	*FAS1*	*FAS1*	*-*	2.3.1.86
*CPAR2_209250*	*COQ5*	*COQ5*	2.1.1.201	*CPAR2_803560*	*GUA1*	*GMPS*	6.3.5.2
*ERG11*	*ERG11*	*CYP51A1*	1.14.14.154	*CPAR2_100620*	*URA7*	*CTPS1*	6.3.4.2
*CPAR2_303080*	*GUK1*	*GUK1*	2.7.4.8	*CPAR2_804900*	*URA1*	*-*	1.3.98.1

Blue: enzymes without any human homolog or drug association. Red: enzymes targeted by drugs currently used to treat *Candida* infections. Green: enzymes with homologs that are currently targeted in the treatment of infections caused by other pathogens.

## Data Availability

All data generated in this study is available in the paper or as supplementary material.

## Supplementary Materials

The following supporting information can be downloaded at: https://www.mdpi.com/article/10.3390/genes13020303/s1, Supplementary file S1, Supplementary file S2.
